# Jamming Recognition Based on Adaptive Feature-Focusing Convolutional Neural Network for Agile Cognitive Radar

**DOI:** 10.3390/s26134296

**Published:** 2026-07-06

**Authors:** Jialei Liu, Jiazhi Ma, Longfei Shi, Zhikang Lin, Yukai Kong, Junxian Chen

**Affiliations:** College of Electronic Science and Technology, National University of Defense Technology, Changsha 410073, China; liujialei@nudt.edu.cn (J.L.); longfei_shi@sina.com.cn (L.S.); z_k.kang@nudt.edu.cn (Z.L.); kongyukai25@nudt.edu.cn (Y.K.); chenjunxian0809@nudt.edu.cn (J.C.)

**Keywords:** jamming recognition, radar waveform parameter agility, adaptive feature-focusing (AFF), mapping relationship, feature scales, 1D-2D feature fusion

## Abstract

**Highlights:**

Faced with the performance trade-off between jamming recognition and agile anti-jamming, this study proposes an adaptive feature-focusing (AFF) network. By pre-training the AFF module, the network establishes a mapping between agile parameters and adaptive feature scales, effectively resolving the feature mismatch caused by parameter agility. Specifically, the AFF module processes real-time high-resolution range profile (HRRP) and short-time Fourier transform (STFT) features, which are then fed into a dual-branch feature fusion network for jamming recognition. This method enables the recognition of multiple typical noise and deception jamming signals using a single pulse without accumulation, making it well-suited for radar jamming recognition under inter-pulse parameter agility.

**What are the main findings?**

- A new formulation of the jamming recognition problem: This paper proposes a framework that captures the trade-off between parameter agility and jamming recognition performance and establishes a corresponding dataset for validation.
- An adaptive feature-focusing (AFF) processing module: It is demonstrated that the AFF module effectively facilitates radar jamming recognition in both parameter-agile and non-agile scenarios.
- A feature fusion paradigm balancing information extraction and computational efficiency: A fusion CNN network incorporating 1D HRRP and 2D STFT features is proposed. These two features provide range scattering characteristics and joint time–frequency components, respectively. Integrated with the AFF module, this network achieves high efficiency and comprehensiveness.

**What are the implications of the main findings?**

- Electromagnetic environment perception and radar jamming recognition.

**Abstract:**

With the advancement of cognitive radar, applying deep neural networks to radar jamming recognition has become an indispensable research direction. However, as a common anti-jamming measure, the agility of radar waveform parameters degrades the effectiveness of jamming recognition, creating a trade-off between jamming recognition and anti-jamming agility. Specifically, for the same type of jamming, radar agility in frequency, pulse width, and bandwidth alters the profile and scale features of the jamming, posing challenges to conventional CNN-based jamming recognition. To address this challenge, this paper proposes an Adaptive Feature-Focusing CNN (AFF-CNN). A pre-trained AFF module is designed to establish a mapping between agile parameters and adaptive feature scales. Operating on time-domain high-resolution range profiles (HRRP) and time–frequency domain short-time Fourier transform (STFT) data, this module calibrates deviations induced by radar inter-pulse parameter agility and enhances the capability of salient signal feature-focusing. Furthermore, a lightweight 1D-2D feature fusion CNN is designed to process these adaptive features and recognize jamming using single-pulse signals, thereby enhancing the network’s adaptability to inter-pulse parameter agility in radar systems. Simulation results demonstrate superior recognition accuracy and generalization capability compared to five comparative approaches, confirming effective adaptation to inter-pulse agility scenarios.

## 1. Introduction

Waveform parameter agility, as an active anti-jamming measure for radars, provides unparalleled advantages in both anti-jamming and multi-function task operations [1,2,3]. Conventionally, inter-pulse agility in multi-dimensional radar signal parameters, such as carrier frequency, bandwidth, and pulse width, enables optimized resource allocation based on target quantity and range [4]. Additionally, through randomized or pseudo-randomized inter-pulse parameter switching, such radars significantly enhance low probability of intercept (LPI) and anti-jamming capabilities [5], while also improving target detection [6] and angle-tracking accuracy [7].

Effective jamming environment perception is essential for dynamic anti-jamming in radars with multi-dimensional agile parameters. Although jamming recognition has advanced considerably, most studies have focused on non-agile waveforms, paying little attention to parameter-agile scenarios. Existing research primarily analyzes jamming signals through transformations across different domains, including the bispectrum [8], frequency [9], time–frequency [10], and wavelet domains [11]. After feature extraction and statistical analysis, the discriminative features are retained and fed into a classifier for recognition. In [12], the proposed method applies Singular Value Decomposition (SVD) to the cyclic spectrum features of signals to identify various jamming types. This approach achieves high recognition accuracy even under low Jamming-to-Signal Ratio (JSR) conditions. In [13], discriminative features are extracted from both the time and frequency domains of jamming signals. A multi-model framework combining Decision Trees (DTs), backpropagation neural networks (BPNNs), and Decision Tree-based Support Vector Machines (DTSVMs) is adopted, achieving robust recognition performance while maintaining computational efficiency. In [14], a radar jamming recognition approach based on fractal dimension and Rényi entropy features was introduced. The fractal dimension characterizes the self-similarity and complexity of signals across the time and frequency domains, while Rényi entropy quantifies signal uncertainty and disorder. By employing Multi-class Support Vector Machines (MSVMs), this approach achieves high-precision classification of active radar jamming signals. Such feature-based methods once dominated this research field [15,16,17] and contributed significantly to the development of jamming recognition. However, such multi-domain feature extraction approaches rely heavily on expert knowledge, and their recognition performance is substantially affected by the quality of manually extracted features.

Recent advances in deep learning have revolutionized radar jamming recognition [18]. Convolutional neural networks (CNNs), known for their generalization capabilities and automated feature extraction [19], significantly outperform traditional feature-based methods in terms of accuracy [20,21,22,23,24]. In [25], researchers used the short-time Fourier transform (STFT) to extract time–frequency features of jamming signals. These features were simply cropped to eliminate redundancy before being fed into CNNs for recognition and classification. The framework in [26] integrates deep neural networks with probabilistic modeling to explore signal modulation patterns under uncertain noise conditions. For unknown jamming type identification, researchers developed an open-world jamming recognition approach based on Siamese neural networks (SNNs) [27]. This approach combines jamming recognition with unsupervised clustering algorithms, enabling simultaneous detection of unknown-class communication jamming and recognition of known-class jamming. Many CNN-based jamming recognition methods have emerged, gradually replacing manual feature engineering. Recent advances have primarily focused on multi-domain feature fusion for jamming recognition. For deception jamming identification in extended target scenarios, a residual neural network based on range–Doppler (RD) and time–frequency (TF) feature fusion was proposed [28]. This approach significantly improves deception jamming classification accuracy by extracting richer jamming features through multi-domain fusion. In [29], researchers developed a transfer learning-based weighted ensemble CNN that processes jamming signals in the TF domain and uses the real (R), imaginary (I), magnitude (M), and phase (P) components of the TF domain signals as separate feature branches. By sharing the model parameters of the real part with other sub-classifiers and applying transfer learning to the four feature branches, this approach achieves high-precision jamming recognition. To address the coexistence of single and composite jamming scenarios, ref. [30] introduced a multi-feature fusion network combining the fractional Fourier transform (FRFT) and STFT. The network demonstrates superior capability in extracting detailed jamming features and achieving accurate recognition by leveraging multi-branch features from the fractional domain of jamming signals. Reference [31] proposes an improved lightweight module named GSE-Net. By fusing features obtained from processing jamming signals with three time–frequency transformation methods, namely STFT, CWT, and SPWVD, a low-complexity recognition network is constructed, achieving a recognition accuracy of over 87% for seven types of compound jamming.

However, none of the above studies have discussed the impact of parameter-agile signals on jamming recognition. It is important to overcome the adverse effects of jamming feature variations caused by parameter-agile signals and to design more efficient networks for jamming recognition. Therefore, the main contributions and innovations of this paper are as follows:

(1) Parameter-agile jamming recognition problem framework and database: To address adversarial scenarios involving rapid radar parameter agility, this paper presents a mathematical modeling of agile-parameter radar signals and their corresponding modulated jamming signals. The constructed dataset captures signal features in the time, frequency, and time–frequency domains under parameter-agile scenarios, thereby providing a validated data foundation for evaluating other recognition methods.

(2) Adaptive feature-focusing (AFF) processing method: To address the impact of agile parameters on signal feature profiles and scales, a functional mapping is established between agile parameters and the scales of the feature extraction window. This mapping is implemented via a pre-trained neural network to effectively mitigate feature misalignment caused by parameter-agile signals, thereby providing high-quality features for multi-feature fusion networks.

(3) Lightweight 1D-2D multi-feature fusion network: Based on 1D time-domain HRRP features and 2D time–frequency domain features, this framework characterizes electromagnetic scattering and simultaneous time–frequency components to enhance jamming recognition. Meanwhile, the fusion network is trained and tested using single-pulse received signals, avoiding transformation methods that require multi-pulse information. This results in stronger adaptability to inter-pulse parameter agility and high computational efficiency.

In fact, end-to-end neural networks with multi-scale branches have been proposed to meet the training requirements of image recognition at different scales. By employing multiple convolutional kernels of different sizes, such networks can partially alleviate the limitations of the fixed receptive field and extract multi-scale features simultaneously.

Nevertheless, this approach has two limitations in the context of those papers. First, although multi-scale convolutional kernels can accommodate inconsistent feature sizes, they may cause performance degradation after multi-scale fusion for fixed-size inputs. Meanwhile, they increase the computational complexity of the network by a factor of 3 to 5. Second, the agile parameter range for pulse width and bandwidth in radar signals can exceed an order of magnitude, whereas the adjustable range of conventional multi-scale convolutional kernels is much narrower. For instance, the typical combination of 3 × 3 and 5 × 5 kernels cannot effectively cover the scale modulation range of agile radar signals.

The innovation of this paper is to decouple the feature preprocessing network from the feature recognition network based on the characteristics of agile radar signal processing. With only a small computational overhead introduced by the lightweight BP-based pre-processing network—which has a much lower computational cost than convolutional networks—superior adaptive feature scaling performance can be achieved.

The paper is organized as follows. Section 2 briefly introduces the mathematical models of inter-pulse parameter-agile radar and jamming signals. Section 3 details the 1D-2D fusion recognition network based on adaptive feature-focusing. Section 4 describes the parameter settings, results, and analysis of the simulation experiments. Finally, Section 5 concludes the paper.

## 2. Signal Model

**Figure 1:**
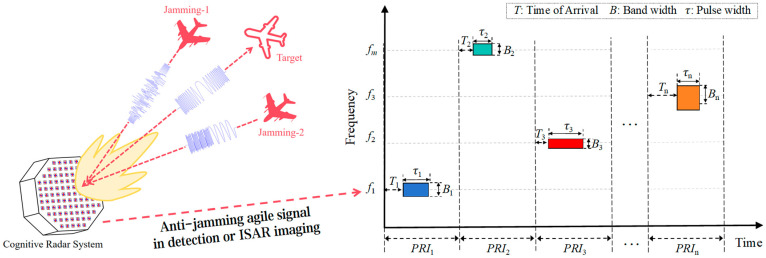
Schematic diagram of a multi-dimensional parameter-agile radar signal.

This study focuses on the efficient modeling and recognition of various jamming types in parameter-agile scenarios. On this basis, this section constructs a modeling scheme for multi-dimensional parameter-agile radar signals as well as active noise and deception jamming signals. The research scenario is illustrated in Figure 1. The cognitive radar system transmits signals with multi-dimensional parameter agility, which are then applied to radar detection or ISAR imaging. Key signal parameters, including carrier frequency, bandwidth, and pulse width, can achieve dynamic agility between pulses. This paper focuses on the effective cognition of electromagnetic jamming even when agile anti-jamming signals are employed.

The proposed AFF-CNN network primarily incorporates two key modalities: 1D HRRP features in the time domain and 2D STFT features in the time–frequency domain. Consequently, this study specifically investigates the impact of multi-dimensional agile radar signals on HRRP and STFT features as follows.

### 2.1. Parameter-Agile Radar Signal Model

Linear Frequency Modulated (LFM) signals, characterized by a large time-bandwidth product, enable radar to achieve a long detection range and high range resolution simultaneously. Hence, this study adopts the LFM signal as transmitted signal. Based on its mathematical definition, with inter-pulse agility in three parameters—namely, carrier frequency, bandwidth, and pulse width—its time-domain expression can be defined as:

(1) $$s(t)=rect\left(\frac{t}{\tau_{i}}\right)\exp\left[j2\pi \left(f_{i}t+\frac{B_{i}t^{2}}{2\tau_{i}}\right)\right]$$

In the equation, $f_{i}$, $\tau_{i}$ and $B_{i}$ denote the agile central frequency, pulse width and bandwidth of the i-th pulse in the radar signal. The rectangular function $rect\left(t/\tau_{i}\right)$ is defined as follows:

(2) $$rect\left(\frac{t}{\tau_{i}}\right)=\left\{\begin{array}{l} 1,\;\left|t-\tau_{i}\right|\leq 1/2\\ 0,\;\mathrm{otherwise}. \end{array}\right.$$

For LFM signals, pulse compression is an effective processing method for maximizing the signal-to-noise ratio (SNR) and enhancing radar system performance. According to matched filter theory, its mathematical expression is:

(3) $$h(t)=s^{*}(\tau_{i}-t)$$

Correspondingly, when the input signal passes through the matched filter, pulse compression is achieved, resulting in a narrow pulse output that significantly improves the radar’s range resolution. This mathematical process can be expressed as follows:

(4) $$\begin{array}{l} s(t)=h(t)⊗s(t)\\ \;\;\;\;\;\;\;=\frac{\sin\left[\pi B_{i}(t-\tau_{i})\left(1-\frac{\left|t-\tau_{i}\right|}{\tau_{i}}\right)\right]}{\pi B_{i}(t-\tau_{i})\left(1-\frac{\left|t-\tau_{i}\right|}{\tau_{i}}\right)}\times \left(1-\frac{\left|t-\tau_{i}\right|}{\tau_{i}}\right) \end{array}$$

From the above, it can be seen that the HRRP result after pulse compression of the LFM radar signal is a sinc function. When $t=\tau_{i}$, the output pulse is at its peak; when $t=\tau_{i}\pm 1/B$, the output pulse is at the first zero crossing, and the main lobe pulse width is 2/B. Furthermore, from the pulse compression mathematical expression of multi-dimensional agile signal, it is evident that the profile and scale of HRRP are only related to the agile pulse width $\tau_{i}$ and bandwidth $B_{i}$ and are independent of the agile frequency. The pulse width mainly reflects the time-domain displacement of the HRRP after pulse compression, while the bandwidth mainly reflects the width and shape differences of the main lobe and side lobes. Both affect the network’s ability to extract profile and detailed features of the signal.

STFT serves as an important basis for analyzing and recognizing the intra-pulse features of different signal types. According to the mathematical definition of STFT, when the input signal is an LFM signal with agile parameters, its expression is as follows:

(5) $$\begin{array}{l} STFT\left(t,f\right)=\int_{-\infty }^{\infty }rect\left(\frac{t}{\tau_{i}}\right)\exp\left[j2\pi \left(f_{i}t+\frac{B_{i}t^{2}}{2\tau_{i}}\right)\right]\;\\ \;\;\;\;\;\;\;\;\;\;\;\;\;\;\;\;\;\;\;\;\;\;\;\;\;\;\;\;\;\;\;\;\;\times w\left(t-\tau \right)\times e^{-j2\pi ft}dt \end{array}$$

In the equation, the first part within the integrand represents the parameter-agile LFM signal, $w\left(t-\tau \right)$ is a segmented window function, typically centered at time t, and $e^{-j2\pi ft}$ is a complex exponential function used for frequency analysis.

From the above, the result of the STFT integral represents the local spectral characteristics of the signal at a specific time and frequency. The scale and profile features of the STFT signal are determined by the pulse width $\tau_{i}$ and bandwidth $B_{i}$. The frequency-dimension position of the STFT signal is determined by the center frequency $f_{i}$. However, the center frequency does not affect the detailed features of the STFT signal and has a smaller impact on STFT signal recognition.

### 2.2. Jamming Signals Model

(1) Noise Amplitude Modulation (NAM) Jamming: NAM jamming is a type of noise jamming that modulates Gaussian white noise onto the bandwidth of the radar LFM signal. It uses high-energy modulated noise to suppress the radar signal, thereby affecting signal detection at the receiver. Its theoretical mathematical expression is as follows:

(6) $$J_{\mathrm{NAM}}=A\left(U_{0}+mn(t)\right)\times \exp\left(2\pi f_{c}t+\phi_{0}\right)$$

where n(t) represents zero-mean Gaussian white noise, $U_{0}$ is the carrier voltage, m is the noise modulation coefficient, $f_{c}$ is the jamming carrier frequency, and $\phi_{0}$ is the phase uniformly distributed over $\left[0,\;2\pi \right]$.

(2) Convolution Noise (CN) Jamming: CN jamming is a convolutional modulation jamming technique based on Digital Radio Frequency Memory (DRFM) technology. It aims to produce a sophisticated noise jamming effect by convolving the delayed radar signal with Gaussian noise, which can suppress the real target signal in both the time and frequency domains. Its theoretical mathematical expression is as follows:

(7) $$J_{\mathrm{CN}}=s(t-\tau )⊗N(t)$$

where “⊗” denotes the convolution operation, and τ is the convolution delay applied to the radar signal.

(3) Multi False-Target (MFT) Jamming: MFT jamming is generated by the jammer through multiple delays, overlaps, and forwarding of the intercepted radar signals to form multiple false targets, with the purpose of deceiving and consuming radar resources. The theoretical mathematical expression is as follows:

(8) $$J_{MFT}(t)=\sum_{n=1}^{N}A_{n}s\left(t-t_{0}-\tau_{n}\right)\times \exp\left[j2\pi \left(f_{0}t+K/2t^{2}\right)\right]\;$$

where $n\left(n\geq 5\right)$ is the number of false targets forwarded by the jammer, $A_{n}$ is the modulation amplitude of the i-th forwarding, and $\tau_{n}$ is the time delay of the i-th false target.

(4) Interrupted Sampling and Repeating (ISR) Jamming: ISR jamming uses a time-division transceiver system, in which the jammer intermittently samples and forwards the radar signal. This method has good real-time performance and can achieve matched filter gain. By sampling and forwarding multiple times within a sampling period, it forms multiple deceptive false targets in the time–frequency domain. The theoretical mathematical expression is as follows:

(9) $$\begin{array}{l} J_{ISR}(t)=\sum_{n=1}^{N}A_{n}rect\left(\left(t-t_{ISR}/2-(n-1)T_{s}\right)/T\right)\;\\ \;\;\;\;\;\;\;\;\;\;\;\;\;\;\;\;\;\;\;\;\;\;\;\times \exp\left[j2\pi \left(f_{0}t+K/2t^{2}\right)\right] \end{array}$$

where T is the radar signal pulse width, $t_{ISR}$ is the jammer’s intermittent sampling pulse width, $T_{s}$ is the intermittent sampling period, and $A_{n}$ is the modulation amplitude of the i-th intermittent sampling and forwarding jamming.

(5) Chopping and Interleaving (CI) Jamming: CI jamming involves the jammer capturing the radar signal, mixing and digitally sampling it, and then storing it in digital memory. The signal is then read out, uniformly sampled at equal intervals, and segmentally replicated. This type of jamming can achieve significant matched filter gain, forming a dense range false target jamming effect against pulse compression radar. The theoretical mathematical expression is as follows:

(10) $$J_{\mathrm{CI}}(t)=\sum_{k=0}^{n-1}A_{k}p\left(t-kT/mn\right)\;$$

(11) $$p(t)=s(t)\left[rect\left(\left(t-\tau_{CI}\right)/\tau_{CI}\right)⊗\sum_{l=0}^{m-1}\delta \left(t-lT_{a}\right)\right]$$

where m is the number of signal pulse sequences sampled by the jammer, n is the number of times each pulse sequence is replicated and forwarded, $\delta \left(\cdot \right)$ is the unit impulse function, $\tau_{CI}$ is the pulse width of the rectangular pulse sequence, and $T_{a}$ is the fundamental period of the rectangular pulse sequence.

(6) Smeared Spectrum (SMSP) Jamming: SMSP jamming involves the jammer capturing the radar signal, mixing it, digitally sampling it, and then storing it in digital memory. Subsequently, through register group shifting and clock frequency modulation, the original radar LFM signal’s frequency modulation rate is altered by a factor of N, and the signal is repeatedly concatenated N times to form a jamming signal with unchanged pulse width. This type of jamming creates false targets in the range dimension and a smeared spectrum in the frequency dimension. The theoretical mathematical expression is as follows:

(12) $$J_{\mathrm{SMSP}}(t)=J_{0}\left(t-t_{d}\right)e^{j2\pi f_{d}t}⊗\sum_{i=1}^{N-1}\delta \;\left(t-iT_{p}/N\right)$$

where N denotes the number of sub-pulse shifts and replications in SMSP jamming, and $t_{d}$ and $f_{d}$ represent the time delay and Doppler frequency modulation of the SMSP jamming, respectively. According to the principle of SMSP jamming signal generation, the clock frequency is adjusted to N times the original frequency to obtain the first sub-pulse signal modulated by the radar signal. The mathematical expression of $J_{0}(t)$ is as follows:

(13) $$J_{0}(t)=\exp\left(j\pi N\frac{B}{T_{p}}t^{2}\right)\;\;0\leq t\leq \frac{T_{p}}{n}$$

### 2.3. Effects of Agile Parameter Modulation

**Figure 2:**
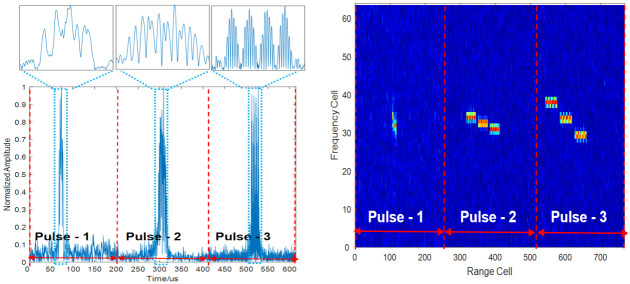
HRRP and STFT features of CI jamming (parameter agility in three pulses).

To analyze the impact of parameter agility on HRRP and STFT features, three groups of signal features with different agile parameters are plotted in Figure 2. It can be seen that due to the influence of parameter agility, although all three pulses are CI jamming sampling instances derived from an LFM signal, significant differences manifest as follows:

In the HRRP feature, the peak of the forwarded target becomes sharper as the signal bandwidth increases, and the range resolution becomes clearer. In the STFT feature, as the signal bandwidth and pulse width increase, the feature profile and scale in the range and frequency dimensions increase significantly. However, for the same type of jamming, the characteristic differences introduced by agile parameters cause a mismatch effect on feature extraction. This effect becomes even more pronounced when the variable waveform signal is modulated by different jamming modes, as the jamming signal characteristics become increasingly inaccurate.

Therefore, designing an adaptive feature scaling module to process received signals is key to improving jamming recognition performance in parameter-agile radar systems. Such a module can maintain high correlation similarity for HRRP and STFT features of the same jamming type, even when the pulse width $\tau_{i}$ and bandwidth $B_{i}$ vary rapidly.

## 3. Adaptive Feature-Focusing CNN Algorithm

**Figure 3:**
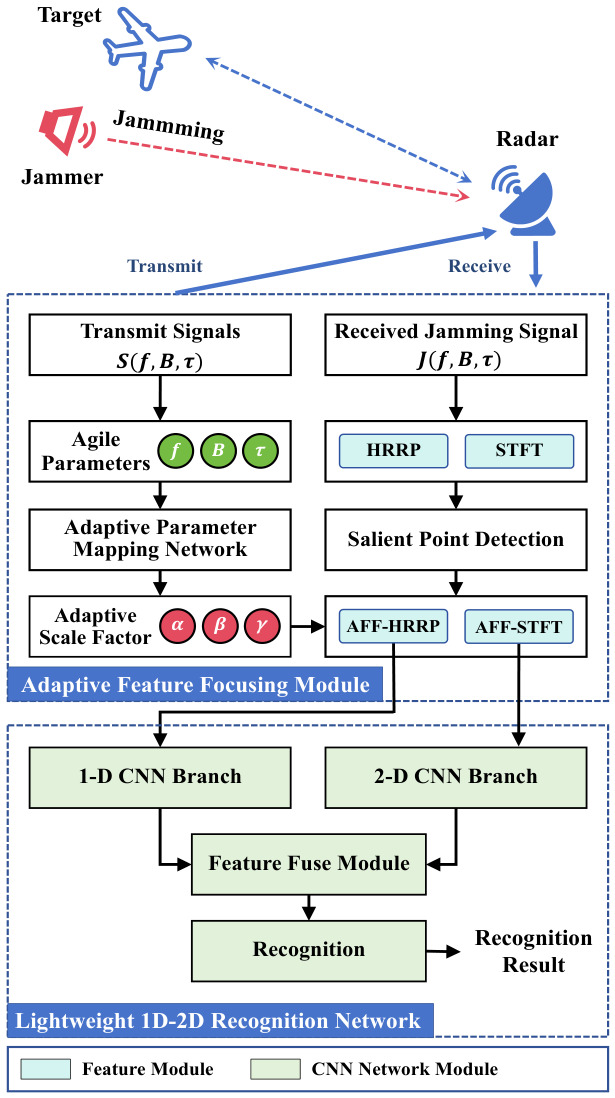
Flowchart of the adaptive feature-focusing convolutional neural network algorithm.

Based on the analysis in Section 2, the HRRP and STFT features vary with agile parameters, which consequently increases the recognition difficulty for the network. To address this challenge, this paper proposes an adaptive feature-focusing CNN network. The network implementation framework is illustrated in Figure 3.

In the application scenario of the network, for radar systems with multi-dimensional parameter agility, the transmitted signal ***S*** varies in waveform parameters such as bandwidth, pulse width, and frequency during target detection. Simultaneously, the jammer intercepts the radar signal and generates modulated jamming signals ***J***, whose characteristics also vary due to transmitted waveform parameters.

In the adaptive feature-focusing processing module, the processing of the transmitted signal ***S*** mainly relies on the adaptive parameter mapping network, which is a multi-layer feedforward neural network; it maps the agile parameters of ***S*** into the scale factors of the feature window. Meanwhile, the received jamming signal ***J*** undergoes pulse compression and STFT processing to generate raw HRRP and STFT signals. These transformed signals then undergo salient point detection, followed by adaptive feature-focusing using the scale factors, ultimately producing AFF-HRRP and AFF-STFT feature signals.

In the lightweight 1D-2D feature fusion recognition network, the features from both the time domain and the time–frequency domain are processed through 1D and 2D CNN branches, respectively, and are then fused to achieve jamming recognition in parameter-agile scenarios.

In the remainder of this section, the specific implementation details of the AFF processing module and the lightweight fusion CNN are introduced.

### 3.1. Adaptive Feature-Focusing (AFF) Module

**Figure 4:**
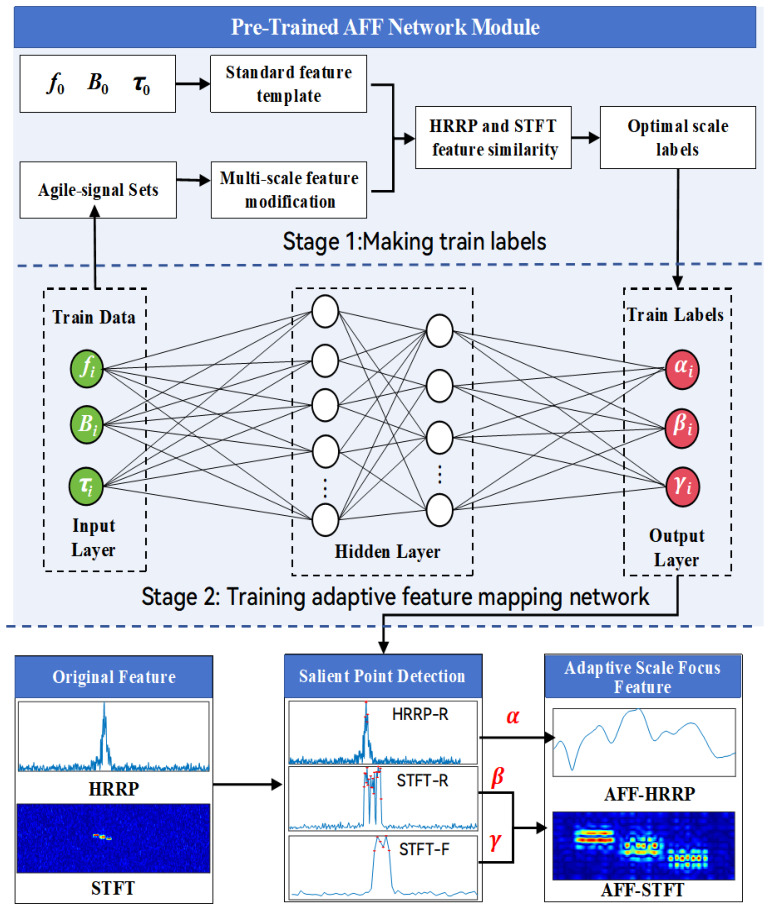
Block diagram of the adaptive feature-focusing module.

The AFF module establishes a mapping relationship between signal agile parameters and feature extraction scale factors through a pre-trained AFF network, which adopts a standard BP neural network with supervised learning. After detecting salient points on the raw HRRP and STFT features, the module performs adaptive-scale window extraction for each salient point and subsequently integrates these extractions to generate focused features. The implementation framework of the AFF module is illustrated in Figure 4.

During the network training stage, multiple groups of jamming pulses with diverse agile parameters are collected to pre-train the parameter mapping network, after which the parameters of the BP network are fixed. In the practical application stage, the radar operates in agile parameter mode. Nevertheless, the agile parameters of the radar itself are known a priori. There is a time delay between signal transmission and reception processing. Within this time delay, the pre-trained network is used to calculate the feature scale factors. Once the radar captures the jamming pulses, adaptive feature-focusing processing can be performed immediately.

Therefore, the AFF network is designed to perform a specific task: mapping radar multi-dimensional agile parameters to the scale factors used in HRRP and STFT feature processing. This can be mathematically expressed as follows:

(14) $$\psi :{ℜ}_{S}^{3}\left(f,B,\tau \right)\to {ℜ}_{L}^{3}\left(\alpha ,\beta ,\gamma \right)$$

In the above formula, ${ℜ}_{S}^{3}$ are the multi-dimensional agile parameter set, ${ℜ}_{L}^{3}$ are the scale factor label set, ${ℜ}_{S}^{3}$ and ${ℜ}_{L}^{3}$ are related through the mapping network, α is the scale factor of the range dimension in HRRP features, and β,γ are the scale factors of range and frequency dimensions in STFT features. The training process of the mapping network consists of two stages.

In the first stage, network training labels are constructed. Based on the generated agile signal set and the template signal, the agile parameters are quantized into multiple states within their respective value ranges. Each state is separately modulated at multiple scales. Subsequently, the similarity is calculated between each modulated signal and the HRRP and STFT features of the labeled template signal at the median range value. The scale factor that yields the maximum similarity is selected as the optimal label.

In the second stage, the AFF network is trained using the agile signal parameter set as the input and the optimal scale factors as the labels, thereby establishing the corresponding parameter mapping. The core objective of the two stages is to construct a mapping between agile parameters and scale factors. Under different agile parameter conditions, the processed feature maps maintain consistency in contour and scale. The detailed mathematical formulation is given as follows:

We take the agile parameters for signal frequency f, bandwidth B and pulse width τ and generate the agile signal sets according to Equation (1) correspondingly. Taking frequency f as an example, the mathematical expression for the agile frequency parameter set is as follows:

(15) $$A(f)=\left\{f^{\left(i\right)}\left|f^{\left(i\right)}=f^{\min}+i\cdot \Delta f,i=0,1,\cdots ,\right.N_{f}-1\right\}$$

In the equation, $f^{\left(i\right)}$ represents the i-th agile frequency value, $f^{\min}$ is the minimum value of the frequency hopping range, Δf is the minimum interval of the frequency jump, and Δf is the total number of agile values. Similarly, the agile parameter sets for bandwidth and pulse width can be obtained, and all combinations of individual agile parameters form the union set of agile parameters.

For comparative purposes, $f_{0}$, $B_{0}$, and $\tau_{0}$, as the standard template signal parameters, are taken as the center values of the frequency, bandwidth, and pulse width agility ranges. The design accounts for the agile range of each parameter so that when the template signal is matched with signals of different agile parameters, the template signal features can serve as a standard to balance the feature differences introduced by the agile parameters. The expression for these values is as follows:

(16) $$(f_{0},B_{0},\tau_{0})=\mathrm{Md}\left(f,B,\tau \right)$$

where $\mathrm{Md}\left(\cdot \right)$ denotes the median operation. Then, based on the template parameters and the agile parameter set, the template signal $S_{0}$ and the agile signal set $S_{i\left(i\geq 1\right)}$ are generated under the LFM signal model. The mathematical expressions are as follows:

(17) $$S_{i}=LFM_{i}(f_{i},B_{i},\tau_{i}),\;i=0,1,\ldots ,N$$

It should be noted that the template signal $S_{0}$ is a single signal, while the agile signal set $S_{i\left(i\geq 1\right)}$ can be taken as N signals corresponding to a set of parameters. After performing PC and STFT operations on $S_{i}$, we can obtain the HRRP features $HR_{i}$ and the time–frequency features $TF_{i}$. Then, projections are made to generate the range dimension projection sequence $I_{i}^{1}$ of $HR_{i}$, as well as the range and frequency dimension projection sequences $I_{i}^{2}$ and $I_{i}^{3}$ of $TF_{i}$. Here, the projection sequences of the HRRP and STFT features can be expressed as $\left\{I_{i}^{k},k=1,2,3\right\}$.

Salient point detection is performed on the projected sequence, and the detected point sequence is denoted as P. The mathematical expression for salient point detection is as follows:

(18) $$P_{i}^{k}=\left\{m\in \{1,2,\ldots ,M^{k}\}|a_{m}>A_{th}\times \mu_{k},a_{m-1}\leq a_{m}\leq a_{m+1},a_{m}\in I_{i}^{k}\right\}$$

where $M^{k}$ represents the number of elements in $I_{i}^{k}$; $a_{m-1}\leq a_{m}\leq a_{m+1}$ indicates that the sequence $a_{m}$ first undergoes local peak detection, where a point is considered a local maximum if it exceeds the detection threshold in the sequence; $\mu_{k}$ is the sequence mean of the projected sequence $I_{i}^{k}$; and $A_{th}$ is the threshold coefficient, which is generally taken as $A_{th}=1.5$.

A threshold equal to 1.5 times the signal mean is commonly used for signal threshold detection. In engineering practice, this coefficient can be chosen from the range of 1.5 to 2. Signal salient point detection adopts a binary decision criterion. It is performed on the projected sequences of HRRP and STFT features in both the range and frequency dimensions. A valid salient point must simultaneously exceed the detection threshold and be a local peak. This detection strategy is designed to retain effective signal characteristics. It can still extract robust detection features even for time-domain piecewise discontinuous jamming signals such as ISR.

Furthermore, based on the value α, β, γ of the scale factor in different dimensions, the sequence of the AFF module of the extracted signal features corresponding to the window sizes of the scale factor in different dimensions can be obtained as:

(19) $$K_{i}^{k}=W\left(P_{i}^{k,1}\left|F^{k}\right.\right)\cup \cdots \cup W\left(P_{i}^{k,M}\left|F^{k}\right.\right)$$

(20) $$W\left(P_{i}^{k,m}\left|F^{k}\right.\right)=\left[P_{i}^{k,m}-F^{k}/2,P_{i}^{k,m}+F^{k}/2\right]$$

where $W\left(P_{i}^{k,m}\left|F^{k}\right.\right)$ represents the rounding operation on the sequence, with $P_{i}^{k,m}$ as the center and $F^{k}$ as the window width. Here, the factor takes the value $F^{k}\in \left\{\alpha ,\beta ,\gamma ,\;\;when\;k=1,2,3\right\}$.

Then, according to the sequence $K_{i}^{k}$, after adaptive scale feature-focusing processing, the features can be defined as AFF-HRRP and AFF-STFT. Their expressions are as follows:

(21) $$\hat{HR_{i}}=sq\left(\left.HR_{i}\right|K_{i}^{1}\right)$$

(22) $$\hat{TF_{i}}=sq\left(\left.TF_{i}\right|K_{i}^{2},K_{i}^{3}\right)$$

where $sq\left(\left.Q\right|K\right)$ represents a new matrix composed of the elements selected from matrix Q, whose subscripts are sequence K. Since actual radar signals typically contain a large number of sampling points, we adopt a signal downsampling strategy to facilitate feature similarity calculation and adjust the feature size.

Next, based on the HRRP and STFT features of multiple sets of agile signals cropped at different scale factors, feature similarity matching is performed with the standard template $\hat{HR_{0}}$ and $\hat{TF_{0}}$. The purpose is to compare different agile parameters and select the optimal scale factor for feature extraction that best matches the standard template features, thereby improving the recognition ability for similar signals. The optimal scale window factor is selected based on the principle of maximum similarity matching, and its mathematical expression is as follows:

(23) $$l_{i}^{\alpha }=\arg\underset{1\leq j\leq L}{\max}\left(corr\left(\hat{HR_{0}},\hat{HR_{i,j}}\right)\right)$$

(24) $$\left\{l_{i}^{\beta },l_{i}^{\gamma }\right\}=\arg\underset{1\leq p,q\leq L}{\max}\left(corr\left(\hat{TF_{0}},\hat{TF_{i,p,q}}\right)\right)$$

In the equation, $corr\left(a,b\right)$ denotes the correlation calculation between the data matrices a and b, and $\arg\underset{j}{\max}\left(R_{j}\right)$ represents the traversal of all values of j to return the maximized parameter $R_{j}$. The returned values are stored in $l_{i}^{\alpha }$, $l_{i}^{\beta }$ and $l_{i}^{\gamma }$ as labels. These labels correspond to the optimal scale extraction windows under the calculation criterion, namely, the optimal extract window α for the HRRP feature and the optimal extract window β,γ for the STFT feature.

By searching and computing the label values, we can establish the optimal features corresponding to a set of agile parameters. The frequency, bandwidth, and pulse width parameters of the agile waveform are used as inputs for network training, and the corresponding optimal scale labels are used as outputs, thereby training a supervised parameter mapping neural network. The expression is as follows:

(25) $$L\left\{l_{i}^{\alpha },l_{i}^{\beta },l_{i}^{\gamma }\right\}=Net\left(f_{i},B_{i},\tau_{i}\right)$$

After network training is completed, the optimal scale factor parameters for any set of agile signal parameters can be computed by the network, and adaptive scale feature extraction can then be performed.

### 3.2. 1D-2D Feature Fusion Network for Single Pulse

**Figure 5:**
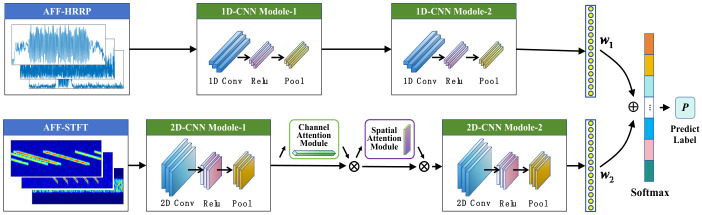
Block diagram of the single-pulse 1D-2D feature fusion jamming recognition network.

This section presents the construction of the AFF-based feature fusion network. The feature extraction and recognition network aims to acquire and train HRRP and STFT feature vectors. Considering the dimensional difference between the two domains, a 1D-2D feature fusion neural network is trained with single-pulse radar received signals. The network can effectively adapt to inter-pulse agile parameters of radar waveforms, as illustrated in Figure 5.

The network consists of three main components: a 1D CNN branch, a 2D CNN branch embedded with an attention module, and a feature fusion module. After AFF processing, the single-pulse adaptive features AFF-HRRP and AFF-STFT are obtained and fed separately into the 1D and 2D feature extraction branches.

In the upper branch for HRRP feature extraction, two cascaded 1D convolutional modules process the jamming signal to extract its temporal features. These modules map the low-dimensional jamming characteristics inherent in the HRRP to a higher-dimensional feature space. Each module comprises a convolutional layer, a rectified linear unit (ReLU) nonlinear activation layer, and a downsampling pooling layer. The two convolutional modules utilize a combination of average pooling and max pooling layers. Average pooling better captures the overall information in the feature map and smooths out noise distortions caused by rapid fluctuations in the temporal waveform. The subsequent max pooling layer retains the salient features of the target after size transformation, further extracting effective information from the signal. The mathematical expressions are as follows:

(26) $$I_{\mathrm{hr}\text{-}1}=\underset{pool}{\mathrm{aver}}\left(\sigma \left(W_{1}\cdot X_{\mathrm{hr}}+b_{1}\right)\right)$$

(27) $$I_{\mathrm{hr}\text{-}2}=\underset{pool}{\max}\left(\sigma \left(W_{2}\cdot I_{\mathrm{hr}\text{-}1}+b_{2}\right)\right)$$

In the equations, $X_{\mathrm{hr}}$ represents the input data of the AFF-HRRP feature in the time domain, $I_{\mathrm{hr}\text{-}1}$ and $I_{\mathrm{hr}\text{-}2}$ correspond to the first- and second-level jamming feature extracted after downsampling, $W_{1}$ and $W_{2}$ are the training weight vectors, while $b_{1}$ and $b_{2}$ are the biases used to enhance the network’s fitting capability, and $\sigma \left(\cdot \right)$ denotes the rectified linear unit activation function ReLU, $\underset{pool}{\mathrm{aver}}\left(\cdot \right)$ and $\underset{pool}{\max}\left(\cdot \right)$ represent the average pooling and max pooling operations, respectively.

In the lower branch for time–frequency domain feature extraction, two cascaded 2D convolutional modules are employed to extract the time–frequency features of the jamming signal. These modules can cover receptive fields of different sizes in the STFT features, thereby effectively extracting jamming information while considering both local and global signal features. Both cascaded 2D convolutional modules utilize ReLU activation layers and downsampling pooling layers. The mathematical expressions are as follows:

(28) $$I_{\mathrm{tf}\text{-}1}=\underset{pool}{\max}\left(\sigma \left(W_{3}\cdot X_{\mathrm{tf}}+b_{3}\right)\right)$$

(29) $$I_{\mathrm{tf}\text{-}2}=\underset{pool}{\max}\left(\sigma \left(W_{4}\cdot I_{\mathrm{tf}\text{-}\mathrm{CBAM}}+b_{4}\right)\right)$$

In the equations, $X_{\mathrm{tf}}$ represents the input data of the AFF-STFT feature in the time–frequency domain, and $I_{\mathrm{tf}}$ corresponds to the jamming features extracted after downsampling by the convolutional module. The definitions of weights, biases, activation functions, and the max pooling layer are the same as mentioned earlier.

Since the recognition of different signal types relies mainly on differences in profiles and detailed features, and since STFT features contain richer information, they are more conducive to the network’s ability to extract discriminative features. To enhance the perception of jamming features in this two-dimensional feature extraction process, a lightweight Convolutional Block Attention Module (CBAM) is inserted to further capture the jamming features.

Specifically, the channel attention mechanism in CBAM effectively captures the regions where jamming exists, while the spatial attention mechanism is used to extract the spatial distribution and shift features of the jamming in the signal image. The overall attention mechanism process in the second branch can be expressed as follows:

(30) $$I_{\mathrm{tf}-\mathrm{CBAM}}=M_{s}\left(M_{c}(I_{tf-1})⊗I_{tf-1}\right)⊗\left(M_{c}(I_{tf-1})⊗I_{tf-1}\right)$$

In the equations, $M_{c}\left(H\right)$ and $M_{s}\left(H\right)$ represent channel attention and spatial attention operations, respectively, and ⊗ denotes element-wise multiplication. The calculation expressions for the channel attention mechanism and the spatial attention mechanism are as follows:

(31) $$M_{c}\left(I\right)=\sigma \left(W_{c_{2}}\left(W_{c_{1}}\left(P_{\mathrm{avg}}^{c}(I)\right)\right)+W_{c_{2}}\left(W_{c_{1}}\left(P_{\max}^{c}(I)\right)\right)\right)$$

(32) $$M_{s}\left(I\right)=\sigma \left(W_{s}\left(\left[P_{\mathrm{avg}}^{s}(I);P_{\max}^{s}(I)\right]\right)\right)$$

where $W_{c_{1}}$ and $W_{c_{2}}$ are the trainable weight vectors of the first and second convolutional layers in the channel attention module, and $W_{s}$ is the trainable weight vector of the convolutional layer in the spatial attention module. $P_{\mathrm{avg}}^{c}$, $P_{\max}^{c}$ and $P_{\mathrm{avg}}^{s}$, $P_{\max}^{s}$ represent the global average pooling and global max pooling of the channel attention module and the spatial attention mechanism module respectively, $\left[\cdot \right]$ denotes the concatenation operation of $P_{\mathrm{avg}}^{s}(I)$ and $P_{\max}^{s}(I)$, and $\sigma \left(\cdot \right)$ represents the Sigmoid activation function.

The information fusion architecture is designed to integrate multi-domain features for jamming recognition, yielding robust and discriminative feature representations. Since each domain captures unique characteristics of radar jamming signals, feature fusion can leverage their complementary advantages to enhance recognition accuracy.

In the information fusion module, the features from the two domains are weighted adaptively to represent their importance. The mathematical expressions for trainable weight-based feature fusion are as follows:

(33) $$F_{\mathrm{fuse}}=\omega_{1}\cdot I_{\mathrm{hr}}+\omega_{2}\cdot I_{\mathrm{tf}}$$

(34) $$P=\mathrm{softmax}\left(F_{\mathrm{fuse}}\right)$$

where $I_{\mathrm{hr}}$ and $I_{\mathrm{tf}}$ are the fully connected layer outputs for the HRRP and STFT features, to guarantee the validity of weighted summation during feature fusion, and features $I_{\mathrm{hr}}$ and $I_{\mathrm{tf}}$ are normalized separately; $\omega_{1}$ and $\omega_{2}$ are the trainable weight scores for the two domains; $F_{\mathrm{fuse}}$ is the result of feature fusion; and P is the score for signal type recognition after feature fusion.

## 4. Experimental Results

In this section, the settings of the agile signal dataset and the corresponding radar jamming dataset are first introduced. Then, the experimental parameter settings of the AFF-CNN algorithm and the comparison algorithms are described. Finally, we simulate and analyze the effects of adaptive feature-focusing extraction and evaluate algorithm performance from multiple aspects.

### 4.1. Datasets

To evaluate the recognition performance of the proposed approach, the paper conducts experiments based on the radar agile signal model and jamming signal model introduced in Section 2. The LFM waveform is selected as the probing signal, and the detailed settings for the signal waveform parameters and jamming parameters are shown in Table 1. The center frequency of the signal is *f*_0_ = 3 GHz, and the sampling rate is *f*_s_ = 40 MHz.

For each instance in the radar received signal *S*_t_, pulse compression is performed to obtain the time-domain HRRP dataset *S*_hr_, where each instance is resized to 1 × 512. The STFT is applied to *S*_t_ to obtain the time–frequency domain dataset *S*_tf_, where the input time-domain instances are divided into 256 segments with 64 points of overlap between adjacent segments. A 16-point Hamming window is selected as the window function for the STFT, and the number of Fourier transform points is 64, resulting in a size of 64 × 256 for each time–frequency instance.

The dataset comprises six jamming types and target signals (detailed in Table 1), with 1000 instances per type, yielding a total of N = 7000 samples. The data was partitioned into training and testing sets with an 80%/20% split, respectively.

### 4.2. Experiment Settings

The proposed AFF-CNN algorithm consists of three branch modules, with the structure and parameters of each shown in Table 2. These modules include convolutional layers, pooling layers, CBAM attention modules, regularization layers, and fully connected layers. Additionally, the model is trained for 40 epochs.

In the pre-training stage of AFF-Net, the variation intervals of carrier frequency, pulse width, and pulse repetition interval are uniformly quantized into 10 agile states with different parameter values, according to the agile parameter ranges defined in Table 1. A total of 1000 samples are generated to cover all states. For the standard template signal, the fixed-parameter signal at the median of the agile range, and $f_{0}=150\;\mathrm{MHz}$, $B_{0}=5\;\mathrm{MHz}$, and $\tau_{0}=50\;\mathrm{us}$ are selected as the benchmark template parameters.

The standard sample signals are processed by adaptive scale focusing. In view of the above size cropping requirement, for the 1 × 512 HRRP feature and 64 × 256 STFT feature, according to the design of the scale window, 1/16 of the total length or width of the image is taken as the initial scale factor, denoted as $l_{0}^{\alpha }=32$, $l_{0}^{\beta }=16$ and $l_{0}^{\gamma }=4$.

Simulations incorporate the agile waveform parameters and jamming models from Section 2. Six typical jamming types and target signals are considered, namely TARGET, NAM, MFT, ISR, CI, CN, and SMSP. The 1D-CNN and 2D-CNN use time-domain and time–frequency domain jamming data, respectively, with parameters as shown in Table 1. The proposed algorithm is compared with the following methods: the time–frequency domain 2D convolutional network [24]; the multi-domain feature fusion CNN with attention mechanism (MDFRCNN) [28]; the multi-feature fusion network based on the time–frequency domain and fractional Fourier transform domain (MFF-Net) [30]; the network fusing features from three time–frequency transformation methods (GSE-Net) [31]; and the multi-weight ensemble CNN based on TF amplitude and phase data (WECNN-TL) [29]. In addition, to verify the effectiveness of integrating the AFF module with lightweight networks, we compared the performance of the proposed 1D-2D fusion network with that of MobileNet [32], which is also a lightweight network.

All experiments were conducted on a computer equipped with a 2.20 GHz CPU, 32.0 GB RAM, and a Quadro RTX 4900 GPU, using Python 3.7 (64-bit). The simulations employ the Monte Carlo method, and all presented results are averaged over 10 independent runs of the algorithm to ensure reliability.

### 4.3. Experiment Results

**Figure 6:**
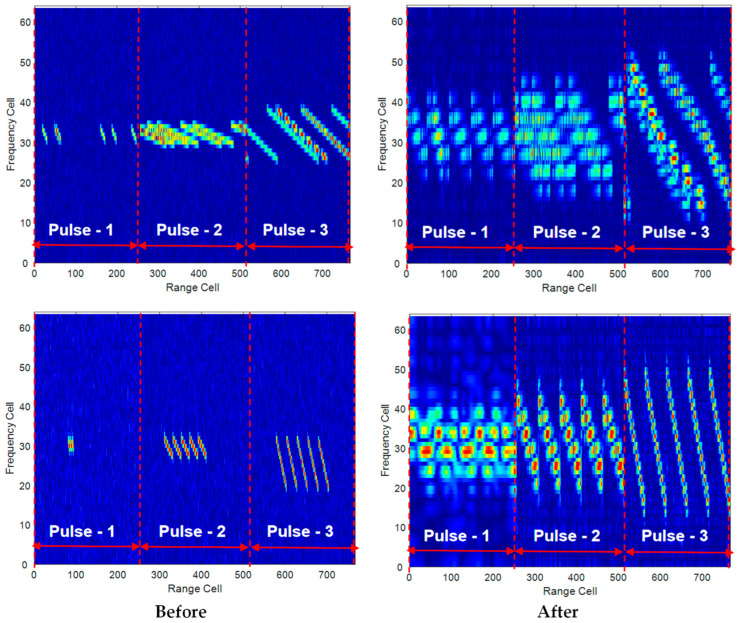
STFT features before and after AFF processing for MFT and SMSP jamming. (Columns 1 and 2 correspond to MFT and SMSP jamming, respectively; the left and right subfigures show features before and after AFF processing).

To compare the performance of different algorithms, this paper analyzes the results from four aspects:

(1) Adaptive Feature-Focusing Processing Effectiveness Analysis: To address the impact of multi-dimensional parameter agility on the feature perturbation of jamming signals, the algorithm first employs the adaptive feature-focusing module to ensure feature consistency and uniformity across varying agile parameters. To evaluate the effectiveness of the AFF module, we selected three distinct parameter-agile pulses for both MFT and SMSP jamming types. Figure 6 visualizes STFT features before and after AFF processing. From the original STFT before AFF processing, due to the effects of bandwidth and pulse width agility, significant variations in STFT features are observed even for the same type of jamming signal. Conventional CNNs perform regional feature extraction using fixed-scale windows, resulting in inconsistent feature representations for identical jamming types, which degrades recognition accuracy. From the AFF-STFT features after AFF processing, the same type of signals can effectively adapt to the influence of parameter variability due to the adaptive window matching, and the signal features maintain good consistency across different groups of varying parameters.

Further quantitative analysis examined the AFF module’s performance under agile parameters. Table 1 details the signal variation ranges. For each parameter, 100 variation values spanning these ranges were selected to form distinct parameter groups. The feature similarity for HRRP and STFT features of various signals after AFF processing is presented in Table 3. For the seven signal types, after AFF processing of HRRP features, the similarity of six jamming signal types improves, while that of SMSP jamming decreases. After AFF processing of STFT features, the similarity of six jamming signal types improves, while that of CI jamming decreases. The statistical average similarity of various signal features before and after AFF processing was calculated. The similarity of HRRP features increased from 0.15 to 0.28, and the similarity of STFT features increased from 0.13 to 0.25. This demonstrates the AFF module approximately doubles feature similarity for identical signal types. Such enhancement contributes to the recognition of parameter-agile signals from the perspective of feature learning.

**Figure 7:**
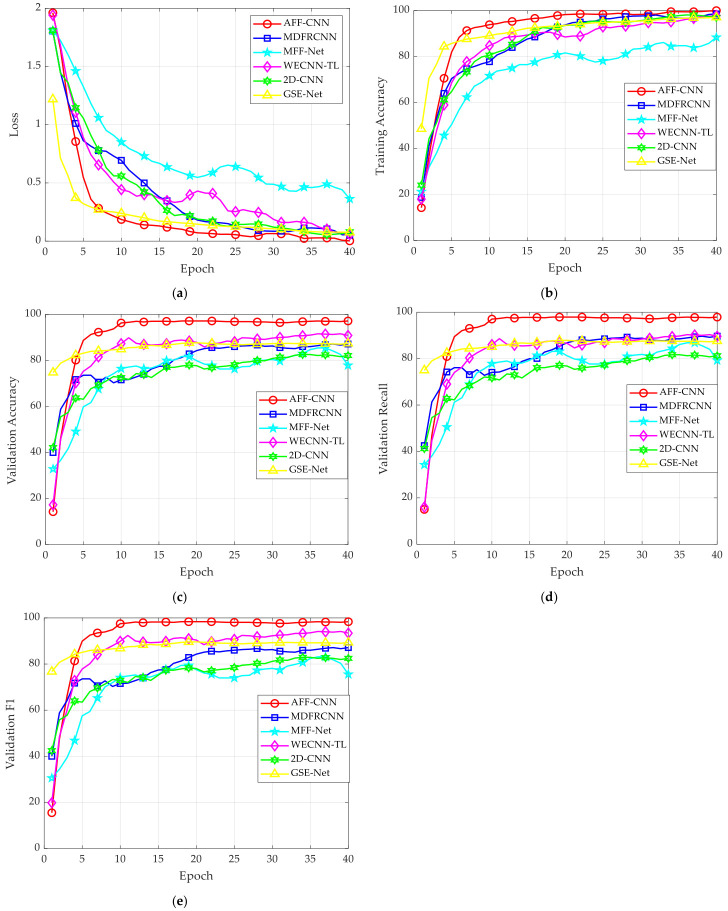
Algorithm comparison results: (**a**) Training loss curves; (**b**) Training accuracy curves; (**c**) Validation accuracy curves; (**d**) Validation recall curves; (**e**) Validation F1 score curves.

(2) Agile Signal Recognition Capability Analysis: After AFF processing, the recognition performance of the proposed AFF-CNN network is compared with that of other networks. The average accuracy for six jamming types and target signals is evaluated at JNR/SNR = 0 dB. The training loss and accuracy curves are shown in Figure 7. Figure 7a depicts the training loss curves. The training loss of AFF-CNN converges to 0.02 and exhibits superior convergence. Other algorithms stagnate near a loss of 0.1, indicating limited feature extraction capability without adaptive processing, which makes it difficult to extract deeper features. Figure 7b shows the training set recognition accuracy curves. All other algorithms, such as MDFRCNN, MFF-Net, WECNN-TL, GSE-Net, and 2D-CNN, exceed 95% accuracy after convergence. AFF-CNN achieves better performance, with an accuracy of 99.76%. Figure 7c illustrates the validation set recognition accuracy curves. Due to signal agility differences, other algorithms show accuracy dropping to 82–88%. AFF-CNN demonstrates stronger adaptability on the validation set and achieves 96.85% accuracy, improving the recognition rate by 10% over the others. Figure 7d,e illustrate the recall and F1 score on the validation set, respectively. Since the training dataset is constructed with a balanced number of samples for each jamming type, the recall and F1 score follow a trend very close to accuracy, with a difference within 1%. Meanwhile, the proposed AFF-CNN maintains a performance advantage of nearly 10%.

**Figure 8:**
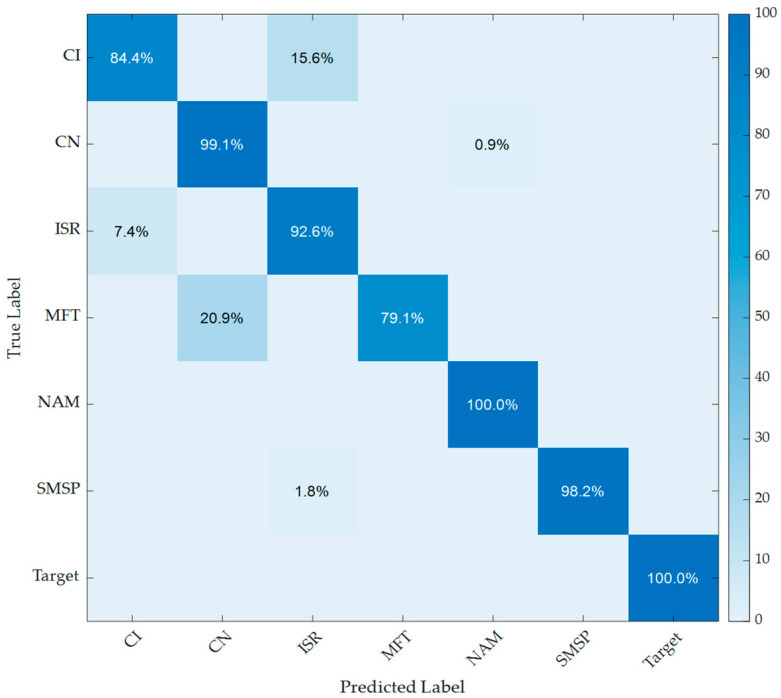
Confusion matrix of jamming recognition accuracy for the proposed AFF-CNN algorithm.

Figure 8 shows the confusion matrix for jamming recognition accuracy of the proposed AFF-CNN algorithm on the validation set. It can be observed that the algorithm achieves high recognition accuracy, close to 100%, for several signal types, including CN, NAM, SMSP, and TARGET. By contrast, the misclassification rates for CI and ISR jamming are relatively high. Both belong to slicing-type jamming, and their main difference lies in whether the sampling interval of signal slicing is prominent, which makes them inherently difficult to distinguish. In addition, when the number of false targets is large, MFT jamming is occasionally misclassified as CN jamming.

**Figure 9:**
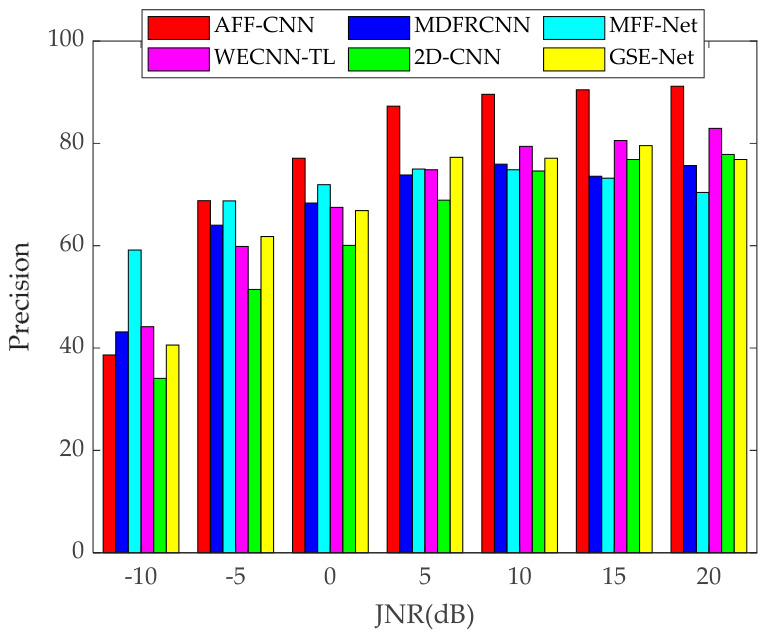
Recognition accuracy under different JNR levels.

(3) Scene Generalization Capability Analysis: During radar anti-jamming, signal strength varies significantly across scenarios due to factors such as radar-target distance, radar-jammer distance, and jamming power modulation. Once the jamming recognition network is trained and its parameters are fixed, the network must be able to generalize across scenarios to handle real-world dynamics. To evaluate algorithm adaptability to signal strength variations, all algorithms were trained at JNR = 0 dB with fixed parameters. Recognition accuracy was then validated using a dataset spanning JNR from −10 dB to 20 dB. The simulation results are shown in Figure 9. It can be seen that recognition accuracy improves significantly with increasing JNR for all five algorithms, confirming that stronger signals aid jamming feature extraction. The four comparison algorithms suffer from degraded performance, with an average recognition accuracy of less than 80%, due to the JNR mismatch between training and validation. The proposed AFF-CNN exhibits superior generalization, maintaining an accuracy of over 90.67% when JNR > 10 dB, thereby demonstrating strong practicality.

**Figure 10:**
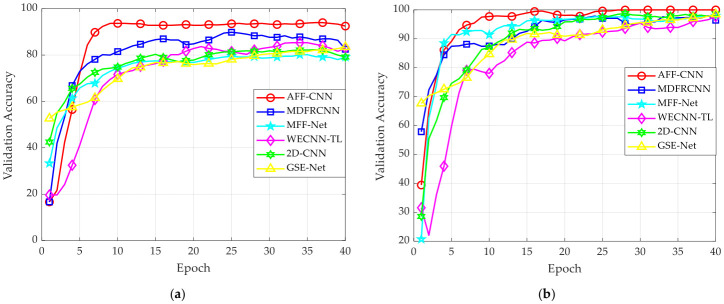
Algorithm comparison results in generalization scenarios: (**a**) Accuracy curves under unequal signal power conditions; (**b**) Accuracy curves under non-agile parameter conditions.

The other two generalization scenarios are the unequal-signal-power scenario and the non-agile parameter scenario, with the algorithm comparison results illustrated in Figure 10. Specifically, Figure 10a corresponds to the unequal-signal-power scenario, where the signal SNR is 0 dB and the jamming JNR is 10 dB. This setup is common and meaningful for scenarios in which jamming is stronger than the target echo. It can be seen that, compared with the equal-power signal scenario in Figure 7c, the overall algorithm performance declined. The recognition accuracy of MFF-Net, WECNN-TL, and 2D-CNN averaged approximately 80%, while MDFRCNN achieved roughly 85%. The proposed AFF-CNN reached 92.16%, demonstrating remarkable superiority. Figure 10b corresponds to the non-agile parameter scenario. Compared with the agile parameter scenario in Figure 7c, the recognition performance of all algorithms improves significantly. This indicates that fixed waveform parameters are more conducive to feature extraction and recognition. The recognition accuracy of all six algorithms exceeds 96%, and the proposed AFF-CNN outperforms its counterparts by an average margin of 1.5%. These results verify that the proposed algorithm remains effective in non-agile parameter scenarios.

(4) Computational Agility and Ablation Study Analysis: As shown in Table 4, the average iteration time across 100 simulation training runs was compared for five algorithms. Each training run used 100 samples per signal type. AFF-CNN and 2D-CNN exhibited the shortest computation times at 2.85 s and 2.97 s, respectively. Although 2D-CNN processes only STFT features, the proposed AFF-CNN fuses both 1D HRRP and 2D STFT features. The low computational burden of 1D HRRP effectively enhances recognition capability and convergence, resulting in AFF-CNN being slightly faster than even the single-domain 2D-CNN. Compared with the MDFRCNN method (fusing STFT and RD features), the MFF-Net method (fusing STFT and FRFT features), the WECNN-TL method (fusing the real and imaginary parts of STFT features), and the GSE-Net (fusing three time–frequency transformation methods), the runtime efficiency of the proposed algorithm is improved by 98.45%, 55.96%, 179.49%, and 35.76%, respectively. Additionally, this comparison accounts only for network recognition time and excludes the time for signal processing and feature generation. Algorithms relying on RD or FRFT features require accumulating multiple pulses or complex transforms, adding extra computation time. In contrast, the HRRP and STFT features in the proposed algorithm can be obtained from a single pulse, making it more computationally efficient and better adapted to scenarios with rapid, multi-dimensional parameter agility between pulses.

**Figure 11:**
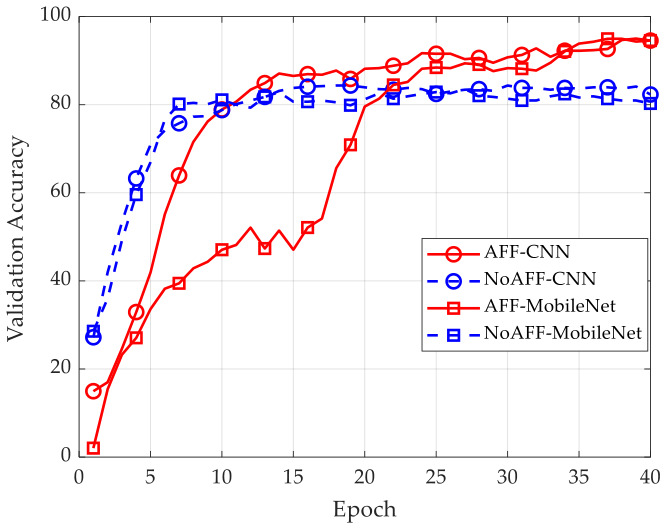
Ablation study results with the AFF module.

In terms of balancing network lightweight design and recognition performance, Figure 11 shows the ablation study results with the AFF module. Before integrating the AFF feature-focusing module, the recognition accuracy of both the feature fusion CNN and MobileNet reached approximately 83%. After integrating the AFF module, their recognition accuracy improved to approximately 95%. As a widely adopted lightweight recognition network, MobileNet has a model size of 1.56 MB, while the model size of the proposed 1D-2D feature fusion CNN network is only 427.7 KB. These results demonstrate that integrating the AFF module with a lightweight network enables excellent jamming recognition performance to be achieved with a smaller network model size.

## 5. Conclusions

Multi-dimensional parameter agility of radar waveforms is a key anti-jamming technique in cognitive radar systems. This paper proposes a novel AFF-CNN algorithm to address the problems of feature misalignment and degraded recognition performance in parameter-agile scenarios. The proposed AFF-CNN aims to mitigate the impacts of parameter agility on HRRP and STFT features, fuse 1D and 2D representations, and recognize jamming signals using a lightweight network architecture.

Simulation results show that, compared with four typical multi-domain fusion algorithms, AFF-CNN maintains a recognition accuracy of at least 93% across diverse scenarios, achieving an average performance improvement of 10% over competing methods. Furthermore, ablation studies confirm that incorporating the AFF module improves the recognition accuracy of the lightweight network by approximately 12%, thereby verifying its critical contribution. The proposed method also exhibits superior recognition performance and generalization capability under varying JNR levels and unequal signal power conditions, and it remains effective in non-agile parameter scenarios.

However, this work does not fully investigate the algorithm’s performance against evolving jamming strategies or modulation styles, nor does it comprehensively evaluate scenarios involving diverse combinations of single and compound jamming. Potential performance degradation may occur under such conditions, which requires further validation in future studies.

## Figures and Tables

**Table 1:** Signal parameter setting.

Signal Type	Parameter	Value Range
TARGET	Agile Bandwidth Agile Pulse width Agile Frequency Relative Delay of each pulse	1–10 MHz 5–100 us 0–300 MHz 0–30 us
NAM	Bandwidth	100 MHz
CN	Bandwidth Noise Bandwidth	1–10 MHz 100 MHz
MFT	Number of false target Interval of false target Bandwidth Pulse width	3~6 0–60 us 1–10 MHz 5–100 us
ISR	Width of slices Number of slices Relative delay of each pulse	2–5 us 4 0–36 us
CI	Width of slices Number of slices Relative delay of each pulse	3–10 us 3 0–36 us
SMSP	Number of jamming sub-pulse Relative delay of each pulse	5 0–36 us

**Table 2:** AFF-CNN network setting.

Branch	Layer	Conv.Kernel	Activation	Pool
Pre-trained AFF-Net	Input	3		
	Hidden	3 × 5 × 15 × 3		
	Output	3		
1-D Convolution Branch	Input	1 × 512		
	Conv.1	(32 × 1) × 16	ReLU	Maxpool(2 × 1)
	Conv.2	(32 × 1) × 10	ReLU	Averpool(2 × 1)
	Flatten	1 × 1040		
	Linear	1040 × 10		
2-D Convolution Branch	Input	64 × 256		
	Conv.1	(3 × 3) × 32	ReLU	Maxpool(2 × 1)
	CBAM	32		
	Conv.2	(3 × 3) × 10	ReLU	Maxpool(2 × 1)
	Flatten	1 × 8680		
	Linear	8680 × 10		
Fuse Module	Linear	10 × 7		
	Output	7 × 1		

**Table 3:** Feature similarity improvement among different parameter groups.

Domain Type	Adaptive Scaling	TAR GET	NAM	MFT	ISR	CI	CN	SMSP	Average
**HRRP**	Before	0.0387	0.0365	0.1904	0.1925	0.0361	0.0390	**0.4750**	0.1457
	**After**	**0.8326**	**0.0375**	**0.3668**	**0.2431**	**0.0370**	**0.3100**	0.0964	**0.2757**
**TF**	Before	0.0858	0.0198	0.0983	0.1170	**0.3361**	0.0778	0.1333	0.1257
	**After**	**0.3098**	**0.0426**	**0.3092**	**0.3107**	0.3319	**0.0998**	**0.3492**	**0.2500**

**Table 4:** Running time of algorithm.

Method	AFF-CNN	MDFRCNN	MFF-Net	WECNN-TL	GSE-Net	2D-CNN
**Time (s)**	2.8522	5.6601	4.4483	7.9717	3.872	2.9742

## Data Availability

The original contributions presented in this study are included in the article. Further inquiries can be directed to the corresponding author.
